# Recent Advances in Polysaccharide-Based Electrospun Nanofibers for Food Safety Detection

**DOI:** 10.3390/s25072220

**Published:** 2025-04-01

**Authors:** Jie Shi, Junjie Tang, Mengfei Zhang, Yingqi Zou, Jie Pang, Chunhua Wu

**Affiliations:** Engineering Research Centre of Fujian-Taiwan Special Marine Food Processing and Nutrition, Ministry of Education, College of Food Science, Fujian Agriculture and Forestry University, Fuzhou 350002, China; 12409019042@fafu.edu.cn (J.S.); 12309019029@fafu.edu.cn (J.T.); wwyyyww115@163.com (M.Z.); 18479577208@163.com (Y.Z.); 000q080031@fafu.edu.cn (J.P.)

**Keywords:** electrospinning, polysaccharides, food safety detection, nanofibers

## Abstract

The continuous advancement of food safety analytical technologies is ensuring food safety and regulatory compliance. Electrospinning, a versatile fabrication platform, has emerged as a transformative methodology in materials science due to its unique capacity to generate nanoscale fibrous architectures with tunable morphologies. When combined with the inherent biodegradability and biocompatibility of polysaccharides, electrospun polysaccharide nanofibers are positioning themselves as crucial components in innovative applications in the fields of food science. This review systematically elucidates the fundamental principles and operational parameters governing electrospinning processes, with particular emphasis on polysaccharide-specific fiber formation mechanisms. Furthermore, it provides a critical analysis of state-of-the-art applications involving representative polysaccharide nanofibers (e.g., starch, chitosan, cellulose, sodium alginate, and others) in food safety detection, highlighting their innovative application in livestock (chicken, pork, beef), aquatic (yellow croaker, *Penaeus vannamei*, *Plectorhynchus cinctus*), fruit and vegetable (olive, peanut, coffee), and dairy (milk) products. The synthesis of current findings not only validates the unique advantages of polysaccharide nanofibers but also establishes new paradigms for advancing rapid, sustainable, and intelligent food safety technologies. This work further proposes a roadmap for translating laboratory innovations into industrial-scale applications while addressing existing technological bottlenecks.

## 1. Introduction

In recent years, accompanied by the progression of the era and the growth in population, the demand for both quantity and quality of food has been increasing significantly. Food has progressively entered phases of industrialization and commercialization. During these processes, the issue of whether food safety can be ensured has garnered public attention. During the processes of food harvesting, processing, packaging, transportation, and storage, factors such as pesticide and antibiotic residues, inadequate washing, improper processing, microbial contamination, and water quality issues may lead to the residual presence of harmful substances in food, ultimately resulting in food safety concerns [1]. Consequently, the detection of these harmful substances in food has consistently been a focal point in the field of food safety research.

With the development of the food industry, the methods for food safety testing have continuously evolved and improved, including techniques such as gas chromatography and liquid chromatography [2], inductively coupled plasma [3], and point-of-care testing [4]. The aforementioned detection methods are accurate and sensitive, yet they impose certain requirements on both the instrumentation and the operator. Consequently, there is a need to explore other potential methodologies.

Electrospinning is a technology with a history exceeding a century, capable of producing micro- to nanoscale fibers and surpassing traditional fiber manufacturing methods [5]. The fiber membranes created through electrospinning possess high specific surface areas, elevated volume ratios, significant porosity, strong electrostatic adsorption capabilities, low weight, and remarkable flexibility. These attributes have led to its extensive applications in advanced fields such as medicine, environmental science, and food technology [6].

Currently, polymer materials used in electrospinning encompass synthetic, natural, and hybrid varieties. Natural polymers, such as polysaccharides, proteins, and lipids, exhibit excellent biocompatibility, low toxicity, and renewability, along with controllable biodegradability, when compared to synthetic polymers like polyurethane, polylactic acid, and polycaprolactone. Among them, polysaccharides are the most abundant and cost-effective bio-based polymers available globally, extractable from a wide range of agricultural and by-product sources [7]. Moreover, due to their biodegradability and biocompatibility, polysaccharides hold significant promise for diverse applications [8]. Electrospun polysaccharide-derived products are now increasingly utilized in biomedical engineering, environmental science, and food applications, primarily in the domains of bioelectronic sensing and food safety detection. Kerwald et al. [9] focused on the in-depth discussion of cellulose-based electrospun nanofibers and summarized the application of cellulose-based electrospun nanofibers in biological and medical engineering. Amorim et al. [10] conducted an in-depth discussion on food packaging fabricated from polysaccharide-based electrospun fibers and their applications. They also explored the prospective integration of natural colorants in polysaccharide electrospun packaging, highlighting the potential for these colorants to function as pH-responsive indicators for detection purposes.

Polysaccharide-based electrospun nanofiber sensors exhibit rapid response and ease of operation, multifunctional integration, and environmental stability in food safety detection. These sensors do not require complex machinery for reading nor specialist handling. Moreover, the finished products have good biocompatibility and biodegradability, ensuring that they do not contaminate food samples during testing and facilitate disposal and recycling post-use.

While significant progress has been made in researching polysaccharide electrospinning across various fields, there is a lack of comprehensive discussions on its application in food safety detection. Therefore, this review aims to fill that gap by focusing on the detection applications of polysaccharide-based electrospun fibers in food products. The article provides an in-depth characterization of electrospinning, with a particular emphasis on the application of commercially available natural polysaccharides [11], including starch, chitosan (CS), cellulose, alginate, and others. Furthermore, it comprehensively details the applications of polysaccharide-based electrospun fibers in food safety detection across livestock (chicken, pork, beef), aquatic (yellow croaker, *Penaeus vannamei*, *Plectorhynchus cinctus*), fruit and vegetable (olive, peanut, coffee), and dairy (milk) products.

## 2. Electrospinning

**Figure 1:**
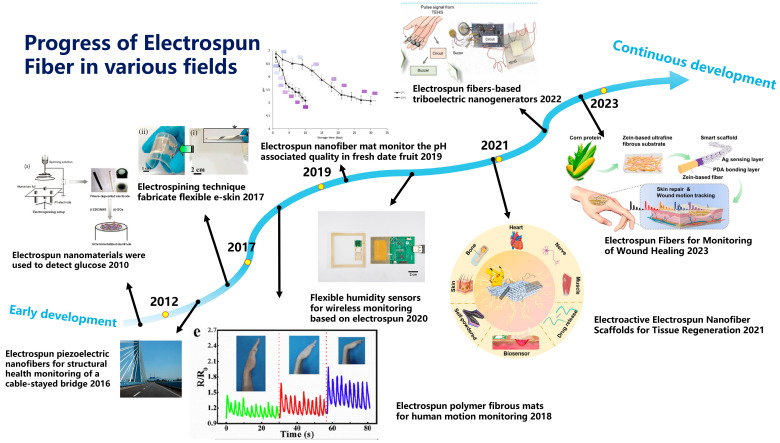
Progress of electrospun fibers in various fields.

**Figure 2:**
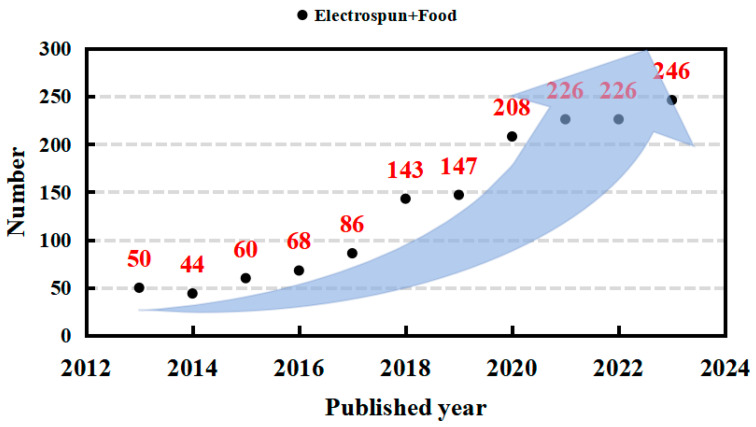
The publication year and the number of papers using electrospun and food as keywords from Web of Science.

**Figure 3:**
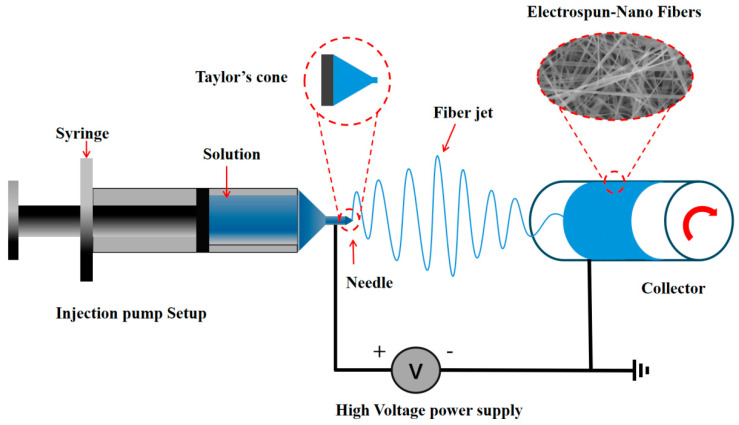
Typical electrospinning apparatus.

Since its inception, electrospinning technology has undergone continuous iterative development, enabling its application across various production practices. Throughout its evolution, electrospinning has demonstrated significant applications in various fields, including biosensing, biomembrane technology, active substance embedding, drug delivery, enzyme immobilization, and the food industry (Figure 1) [12,13,14,15,16,17,18,19,20]. In particular, the research value of electrospinning technology within the food industry has garnered increasing recognition. This is evident from the notable rise in publications containing the keywords “food” and “electrospinning” over the past decade, reflecting a growing convergence of electrospinning innovations with food science applications (Figure 2). The basic setup of an electrospinning device is depicted in Figure 3 [21]. This device comprises a high-voltage power supply, an injection pump, and a collector. During operation, the direction of fiber movement is determined by the polarity of the current; typically, liquid fibers move from the positive electrode to the negative electrode, with the device configured either horizontally or vertically. When activated, the injection pump delivers the spinning solution at a controlled and steady rate, causing it to extrude from the tip of the metal needle [22]. Due to the high viscosity of the spinning solution and gravitational forces, a hemispherical droplet forms at the metal needle tip. Under the influence of the electrostatic field, this droplet is stretched into a conical shape known as the Taylor cone. The spinning solution is then ejected from the tip of the Taylor cone toward the collector. As the solution travels towards the collector, the solvent evaporates rapidly, resulting in the formation of a uniform nanofiber film that solidifies on the collector [21]. Electrospinning can be categorized into various types based on the configuration of the injection pump, collector, and the nature of the spinning solution [8].

## 3. Factors Influencing Electrospinning of Polysaccharides

### 3.1. Process Parameters

The outcomes of the electrospinning process are influenced by multiple factors, which can be primarily categorized into the electrospinning parameters, the properties of the spinning solution, and environmental conditions. Each of these variables can significantly impact the results of electrospinning (Table 1). In the electrospinning process involving polysaccharides, it is essential to comprehend and adjust these parameters effectively. Under the influence of an electrostatic field, the spinning solution forms a Taylor cone. When the applied voltage surpasses a critical threshold, the tip of the Taylor cone releases the spinning solution. This critical voltage varies depending on the specific spinning solution system utilized [21,23,24]. Notably, the relationship between voltage and performance may not always be linear for different polymer spinning solutions.

The feed flow rate refers to the volume of the spinning solution being delivered to the injection needle, while the flow velocity indicates the speed at which the spinning solution is pushed towards the needle. The pumping speed of the spinning solution interacts with the injection needle diameter and the electric field voltage to stabilize the Taylor cone, ultimately influencing the quality of the electrospinning process. Increasing the feed flow rate, while maintaining a stable Taylor cone, can enhance the diameter of the nanofibers to some extent. Research conducted by Amiri et al. [23] demonstrated that, with a specific ratio of CS to gelatin polymer at a fixed voltage and spinning distance, increasing the flow rate from 0.5 mL/h to 1.5 mL/h resulted in a fiber diameter increase from 253.28 ± 39.05 nm to 282.50 ± 42.06 nm (*p* = 0.035).

The spinning distance, defined as the distance between the syringe needle and the collector, is critical for allowing the charged spinning solution to form nanofibers. A shorter spinning distance results in the spinning solution remaining within the electric field for a limited duration, which can impede adequate solvent evaporation. In scenarios where the spinning solution has a high solvent content, this can lead to the dissolution of the nanofiber film already formed on the collector, resulting in suboptimal electrospinning outcomes. Conversely, an extended spinning distance facilitates the production of longer and thinner nanofibers. Therefore, it is imperative to continuously optimize the spinning distance throughout the electrospinning process.

### 3.2. Properties of Polysaccharides Solution

The spinning solution also has a significant impact on the electrospinning results. The main influencing variables include the properties of the spinning solution itself, viscosity, electrical conductivity, and solution composition. In the electrospinning of polysaccharides, taking starch as an example, starch is composed of amylose and amylopectin, both of which are made up of α-D-glucose units, but amylopectin accounts for 70–85% of starch. Studies have shown that amylose has good electrospinning properties due to its tendency to entangle, while amylopectin has the opposite effect, making the entanglement and formation of fibers more difficult [25]. Therefore, using high-amylose starch for electrospinning is a relatively successful method to improve the properties of fibers [26]. There are also methods of mixing polysaccharides with other substances to improve the properties of fibers. For example, NaCl [27], polyethylene oxide (PEO) [28], and trifluoroacetic acid [29].

### 3.3. Environmental Parameters

Among the various factors influencing the electrospinning of polysaccharides, temperature and humidity are frequently overlooked. These environmental variables are critical not only during the spinning process but also for the quality and characteristics of the resultant fibers. Generally, elevated environmental temperatures tend to enhance the outcomes of electrospinning [30,31,32], while increased humidity is predominantly detrimental and should be minimized whenever possible [33]. Therefore, maintaining low humidity levels is essential to ensure optimal electrospinning outcomes and fiber quality.

**Table 1:** The influence of different electrospinning parameters on the results.

	Polysaccharide Polymer	Solvent System	Variable Parameter	Effect on Fiber Morphology	Account	References
Process parameters	CS-Gelatin	Acetic acid	↑High voltage	Diameter decrease	The flow rate and spinning distance are constant, the voltage is increased, and the fiber diameter is increased	Nafise Amiri [23]
	Cellulose acetate (CA)	Acetone	↑High voltage	Diameter decrease	The spinning distance is constant, decreasing or increasing the voltage at 12 kV will reduce the fiber diameter	Nicole Angel [21]
	CA/Ethyl cellulose	Acetone-DMF	↑High voltage	Diameter increase	The spinning distance is constant and the diameter of nanofibers increases with the increase of voltage	Abdallah Refate [24]
	CS-Gelatin	Acetic acid	↑Feed flow rate	Diameter increase	The voltage is constant and the diameter of the nanofibers increases with the increase of the flow rate	Nafise Amiri [23]
Solution parameters	CS/PEO	Phosphoric acid	Add Polymer	More stable	The spinning stability of CS was improved by electrospinning by blending CS with PEO	Wei Pan [28]
	SA/PEO/CaCl_2_/Glutaraldehyde/trifluoroacetic acid	Water	Add Polymer	More stable	SA was blended with other polymers for electrospinning to improve spinning stability	Qian Wang [29]
	CA/bark oil/clove bud oil	Acetone and dimethyl formaldehyde	Add Polymer	More balanced	The blending of cellulose and essential oil improves the evenness of spinning fibers	Maheshika Sethunga [34]
	CS	Acetic acid and trifluoroacetic acid	Add NaCl	More stable	Adding NaCl to CS solution can increase the conductivity and decrease the viscosity	Hengjie Su [27]
	CS/PEO	Acetic acid	Add NaCl	More stable	Adding NaCl to CS/PEO solution can increase the conductivity and decrease the viscosity	Varnaitė-Žuravliova [35]
Environmental Parameters	CS	trifluoroacetic acid and dichloromethane	↑Temperature	More balanced	The nanofibers are most uniform at 32 °C	Dan-Thuy Van-Phan [30]
	CA	Acetone and dimethyl formamide	↑Temperature	Load increase	Increasing the temperature from 22 °C to 52 °C can increase the yield	Awais Khatri [31]
	CA/Polyethylene	Acetone	↑Temperature	More stable	CA/PEI films show an increase in thermal stability	Jinsoo Yoon [32]
	Silk	CaCl_2_/H_2_O/EtOH	↑Humidity	Diameter decrease	The higher the humidity, the worse the fiber performance	Bo Kyung Park [33]

## 4. Electrospinning of Polysaccharide

Polysaccharides have emerged as promising materials for electrospinning due to their abundance, biodegradability, non-toxicity, and versatile biological activities. Key polysaccharide materials used in electrospinning include starch, CS, cellulose and sodium alginate.

### 4.1. Starch

Starch is a high-molecular-weight carbohydrate composed of amylose and amylopectin molecules. Amylose is a linear polymer composed of glucose units linked by α-(1,4) glycosidic bonds, while amylopectin is a highly branched polymer composed of linear chains interconnected by α-(1,6) glycosidic bonds. Starch is also a multifunctional biomaterial with low cost, hydrophilicity, and non-toxicity. Starch is abundant in nature and is an essential polymer for living organisms, being the main source of energy and nutrition for humans and animals. Starch is widely used in food and related research. The inherent properties of this polymer render it ideally suited for diverse applications, including the production of nanomaterials. Electrospinning is employed to create fibers characterized by a high surface area-to-volume ratio, which facilitates the encapsulation of bioactive compounds [36]. In recent years, starch electrospinning has gradually been applied in food preservation. Huang et al. [37] integrated cinnamon essential oil into electrospun octenylsuccinic starch–pullulan nanofiber mats. These mats demonstrated varying degrees of inhibition against *Staphylococcus aureus*, *Escherichia coli*, and *Aspergillus flavus*, with notable effects on *Staphylococcus aureus.* This study highlights the high loading capacity and bioactivity of starch in electrospinning technology. The nanofiber mats effectively inhibited the growth of *Staphylococcus aureus*, *Escherichia coli*, and *Aspergillus flavus*. This work supports the development of starch-based electrospun nanofibers for applications in food safety detection, providing an effective means for identifying microbial contaminants.

### 4.2. Chitosan/Chitin

CS is a polysaccharide obtained through the deacetylation of chitin. Among natural polysaccharides, CS is the only alkaline polysaccharide and is the second most abundant polysaccharide, following cellulose. It is abundantly available in nature, contributing to its low cost. CS is sourced from the shells and claws of crustaceans, as well as from certain fungi and algae. It possesses several advantageous properties, including biocompatibility, antimicrobial activity, and non-toxicity. CS fibers produced via electrospinning exhibit high specific surface areas and porosity, highlighting the superior characteristics of the material. CS nanofibers possess properties that enable the effective capture of particulate contaminants in food, thereby facilitating food safety testing. Kandeh et al. [38] have developed a novel composite material using electrospinning technology, consisting of polyvinyl alcohol (PVA), citric acid, CS, and aloe vera gel. This material was evaluated for its ability to simultaneously monitor pesticides in both water and food, demonstrating acceptable recovery rates ranging from 86.3% to 96.8%.

### 4.3. Cellulose

Cellulose is the most widely distributed and abundant polysaccharide in nature [39]. It has high physical strength and biocompatibility. The main structural bond in cellulose is the β-(1-4) d-pyran glucose residue, and the β-sheet symmetry of this molecule includes intramolecular and intermolecular hydrogen bonds. At room temperature, cellulose is insoluble in water and common organic solvents such as ethanol and ether. Generally, cellulose is very stable. Usually, cellulose is used in electrospinning through composite or modification methods. Alfonso et al. [40] utilized castor oil and cellulose acetate propionate (CAp) as raw materials for their study. They hypothesized that the acetyl and propionyl groups in CAp would provide chemical compatibility with castor oil, thus facilitating the production of electrospun nanostructures. These structures, in turn, could enhance physical stability by creating diverse morphologies. Due to its excellent biocompatibility, Zhou et al. [41] developed a highly specific electrospun chalcone derivative grafted ethylcellulose fluorescent probe for the detection of Al^3+^ in food. Elevated levels of Al^3+^ can damage the hippocampus and affect the nervous system of organisms and are closely associated with the onset of Alzheimer’s disease. After HTC (3′-hydroxy-4′-(3-(2,4,6-trimethoxyphenyl) acryloyl)-[1,1′-biphenyl]-4-carboxylic acid) esterification with hydroxyethyl cellulose, the solubility of the compound decreases, leading to aggregation in water. This aggregation inhibits non-radiative decay, thereby enhancing fluorescence. Consequently, using ethyl cellulose as a material in electrospinning enhances the accuracy of Al^3+^ detection in food safety testing.

### 4.4. Sodium Alginate

SA is a natural anionic polysaccharide isolated from brown algae. Due to its low cytotoxicity, biodegradability, biocompatibility, and mucosal adhesiveness, it is widely used for wound healing promotion and waste adsorption. Zhao et al. [42] successfully prepared a zwitterionic composite sponge by combining CS with electrospun SA nanofibers using a freeze-drying method. This sponge has a rich functional group and an excellent microstructure with interconnected pores and nanofibers, which is beneficial for improving adsorption capacity. It has a considerable simultaneous adsorption capacity for binary systems containing anionic and cationic dyes in dye wastewater. This study demonstrates the strong adsorption capabilities of electrospun sodium alginate nanofibers, highlighting their potential for research in food safety testing. Li et al. [43] have developed a novel electrospun nanofiber film composed of SA/MXene, which effectively adsorbs methylene blue from wastewater. After 10 cycles of reuse, the adsorption capacity and removal efficiency of methylene blue remain above 95%. This demonstration of methylene blue adsorption underscores the significant potential of nanofibers in detecting aquatic food contaminants.

### 4.5. Pullulan

Pullulan is a water-soluble microbial polysaccharide produced by fermentation of Aureobasidium pullulans, composed of α-(1,6)-linked maltotriose units connected by α-(1,4) glycosidic bonds to three pyranose glucose units. It has good film-forming properties, high plasticity, and biodegradability. Pullulan can be used to make food packaging, but its hydrophobicity, antibacterial activity, and mechanical strength still need to be improved. The combination of soy protein amyloid fibrils and pullulan can effectively enhance the mechanical strength and hydrophobicity of pullulan composite films [44]. Although pullulan has good spinnability, it is relatively difficult to produce micron-sized fibers. Amjadi et al. [45] first developed and characterized zein/pullulan electrospun nanofibers loaded with carvone. They found that zein/pullulan nanofibers have a uniform and bead-free morphology, as well as the lowest average fiber diameter (508 ± 159 nm). Jiang et al. [46] have developed a pH-sensitive sensor based on electrospun nanofiber membranes of pullulan/polyvinyl alcohol incorporated with bayberry pomace anthocyanins. This sensor responds to total volatile basic nitrogen (TVB-N) levels in the environment, indicating the freshness of *Penaeus vanname* through color changes detectable by the naked eye. This approach offers a practical solution for assessing seafood freshness in line with food safety standards. However, the firm’s performance is limited in high-humidity environments due to the high water solubility of pullulan. Enhancing the hydrophobic properties of pullulan can facilitate the development of a broader range of applications for this material.

### 4.6. Dextran

Dextran (DEX) is one of the bacterial polysaccharides, composed of α-(1,6)-linked pyran glucose units and side chains connected through α-(1,4) and α-(1,6) bonds. It can be produced by bacteria cultured in sucrose solutions under the catalysis of DEX sucrose enzymes and exists naturally in foods (such as honey and syrup). DEX is a water-soluble polymer, and it is also necessary to adjust it to maintain its mechanical properties. Phulmogare et al. [47] studied the effect of increasing the concentration of fucoidan (FD) on the characteristics of electrospun PVA/DEX nanofibers. Kenawy et al. [48] developed electrospun PVA/DEX nanofibers for wound-healing applications. These nanofibers, cross-linked with citric acid and loaded with sodium ampicillin, were prepared through electrospinning. This study demonstrates that electrospun DEX nanofibers possess desirable cross-linking load characteristics and biocompatibility, highlighting their potential for application in load sensors and pollutant capture.

### 4.7. Hyaluronic Acid

Hyaluronic acid (HA) is a naturally occurring anionic glycosaminoglycan in the human body, composed of alternating disaccharide units of β-(1,4)-D-glucuronic acid and β-(1,3)-N-acetyl-D-glucosamine, and is widely distributed in connective tissues, eyes, and skin throughout the body. HA exhibits many favorable properties, such as biocompatibility, unique physicochemical properties, and biodegradability. However, HA has poor electrospinning performance in water-based solutions. Gruppuso et al. [49] synthesized a polysaccharide-based electrospun wound dressing using HA, lactose-modified CS, and PEO as the main components. However, the nanofibers were unstable in water, so the ability of two new cross-linking methods, methacrylic anhydride and carbodiimide, to stabilize the nanofiber structure was tested. The study achieved favorable results, enabling hyaluronic acid to be effectively used in electrospinning processes.

Yang et al. [50] developed a multifunctional facial mask using electrospun nanofiber mats based on hyaluronic acid and silk fibroin, loaded with protocatechuic anthocyanins and collagen peptides. Although the study did not ultimately focus on food safety detection, it closely aligns with Jiang’s research [46] on pH chromogenic sensor-based food safety testing. This indicates that electrospun nanofibers of hyaluronic acid, as a polysaccharide base, possess substantial potential.

### 4.8. Cyclodextrin

Cyclodextrin (CD) is a general term for a series of cyclic oligosaccharides produced from amylose by CD glucosyltransferase from bacillus. It usually contains 6 to 12 D-pyran glucose units. CD has a special cyclic structure and is non-toxic and environmentally friendly. CD exhibit an exceptional capability to form stable non-covalent complexes with small molecules, significantly enhancing their solubility, stability, and availability, thereby achieving encapsulation efficiencies previously unattainable. CD have been demonstrated to improve the spinnability of formulations based on biopolymers due to their propensity to form supramolecular structures with these polymers. This interaction reduces or even eliminates the need for synthetic co-spinning agents [51]. Consequently, CD enhance the mechanical properties of electrospun products used in food safety detection, and possess substantial potential for future development.

## 5. Application of Polysaccharide-Based Electrospun Nanofibers in Food Safety Detection

**Figure 4:**
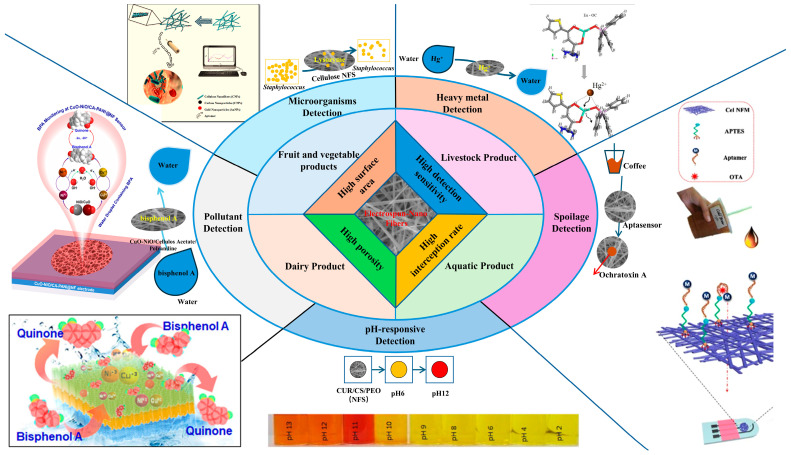
Applications of polysaccharide-based electrospun nanofibers in food safety detection.

Food contamination, a critical challenge in the food industry, predominantly arises from protein denaturation, lipid oxidation, and microbial contamination. These deteriorative processes occurring during processing, storage, and transportation can lead to foodborne illnesses if undetected. Recent advancements in smart technologies have highlighted the potential of polysaccharide-based electrospun nanofibers as innovative sensing platforms. This section systematically examines their application in detecting livestock products, aquatic products, and other foods (Figure 4) [52,53,54,55,56], while addressing current challenges and prospects in applications.

### 5.1. Livestock Products

Electrospun polysaccharide-based nanofibers exhibit exceptional substrate properties for sensor integration due to their high surface-to-volume ratio and interconnected porous architecture. These characteristics enhance analyte interaction, thereby improving detection sensitivity. A seminal study by Bekhit et al. [57] established total volatile TVB-N as a key spoilage indicator in meat products. Generally, an increase in TVB-N content signals a deterioration in meat quality. Yildiz et al. [52] developed a colorimetric pH sensor through the electrospinning of CS/PEO matrices incorporating curcumin (CR) to monitor the spoilage of chicken breast. The introduction of CR endows the film with food safety detection capabilities, as pH changes induced by TVB-N levels in the storage environment lead to color changes in CR, thus exhibiting a sensing function. This method of detecting food safety through pH changes has demonstrated promising results. In other studies, Li et al. [58] produced electrospun nanofiber films using CS/PVA with mulberry anthocyanins(ATH), and Guo et al. [59] developed a novel intelligent dual-layer fiber mat combining pullulan-purple sweet potato extract (PL-PSPE) with corn zein-glycerol-carvacrol (ZN-GL-CA) for pork freshness assessment. These studies similarly employed the same principles to develop polysaccharide-based electrospun nanofiber films with capabilities for detecting poultry meat safety.

Moreover, polysaccharide-based electrospun films, due to their high biocompatibility and electron transfer capability, are often utilized in electrochemical sensing. Rehman et al. [60] developed an electrochemical a clenbuterol (CLN) detection platform using conductive CA–polyaniline electrospun fibers embedded with Co nanoparticle-decorated N-doped porous carbon (CN-CoNPs). Machine learning (ML) optimization further enhanced sensor performance, achieving remarkable amperometric detection limits in beef matrices. This integration of nanomaterials with computational analytics exemplifies the evolution of intelligent detection systems.

### 5.2. Aquatic Products

Aquatic food safety monitoring shares fundamental principles with meat systems, particularly regarding TVB-N accumulation during spoilage. Utilizing this critical indicator, polysaccharide-based electrospun nanofibers serve as a vital method for assessing the safety of aquatic foods, with pH colorimetric sensors being particularly significant. The deterioration of aquatic food quality is often challenging to observe visually; thus, employing pH colorimetric sensors represents an intuitive and efficient choice. Wang et al. [61] developed a smart pH colorimetric sensor by combining carrageenan, CMC, and rose ATH. In practical applications, the sensor was applied to assess the freshness of yellow croaker stored at 4 °C, demonstrating a color change from pink to yellow upon spoilage. The indicator film exhibited high sensitivity to changes in the freshness of the yellow croaker. Furthermore, the polysaccharide-based electrospun nanofibers not only served as the sensing substrate but also provided protection and antibacterial effects against the target analytes. Jiang et al. [46] have developed a pH colorimetric sensor capable of indicating the freshness of *Penaeus vannamei* by incorporating myricetin-based anthocyanins into electrospun nanofibrous films made from pullulan polysaccharide and PVA. This sensor operates by responding to the TVB-N content in the environment. Duan et al. [62] developed electrospun nanofibers based on pullulan/CS nanofibers (PCN), incorporating CR and ATH. In food safety testing experiments, as the quality of *Plectorhynchus cinctus* deteriorated, the PCN/CR/ATH nanofibers displayed a significant color response, transitioning from pink to powder blue, thus validating their pH sensing capabilities. Additionally, antibacterial tests confirmed that polysaccharide-based nanofibers inhibited the growth of Staphylococcus aureus and Escherichia coli. Yurova et al. [63] have demonstrated a more convenient approach for biogenic amine detection by fabricating test paper from electrospun nanofibers of CA. This method not only reduces the cost of detection but also allows for widespread application.

Beyond spoilage detection, environmental contaminant detection is also a critical aspect of food safety detection for aquatic products. Contaminants in water bodies can transfer through the food chain, impacting aquatic foods. Monitoring pollutants at their source can reduce the costs associated with individual testing of specific aquatic products. Common contaminants include heavy metals (such as mercury and cadmium) and other environmental toxins. Polysaccharide-based electrospun fibers can effectively capture and detect these hazardous substances. Fluorescence detection methods are particularly effective in food safety testing. Zhang et al. [53] developed a novel luminescent europium/CA nanofiber (Eu/CA NFs), establishing a correlation between the fluorescence intensity of Eu/CA NFs and the concentration of Hg^2+^, enabling the detection of trace levels of Hg^2+^ in various aquatic environments. Moreover, these nanofibers are reusable. In a similar vein of research, Teodoro et al. [64] have developed a sensor made from electrospun nanofibers of cellulose nanocrystals/reduced graphene oxide and polyamide, capable of detecting Hg^2+^ in aquatic environments. This sensor can detect Hg^2+^ within a concentration range of 2.5–200 μM, with a low detection limit of 5.2 nM. Notably, the developed sensor demonstrates selectivity for Hg^2+^ even in the presence of interfering metals such as Cd^2+^, Pb^2+^, and Cu^2+^. Shoukat et al. [54] created an efficient electrochemical sensor based on CuO-NiO coated CA/polyaniline electrospun nanofibers for the sensitive and selective monitoring of A (BPA). The nanoscale structure of polysaccharide-based nanofibers significantly enhances detection limits, allowing for the rapid analysis of trace pollutants and achieving low detection limits (0.6 nM), a wide linear range (2–100 nM), and high selectivity. The designed electrodes have successfully been applied for the high-precision monitoring of BPA in bottled water and can also be utilized for BPA detection in aquatic environments and aquatic foods.

In research focused on convenient detection methods, Wu et al. [65] explored a sensor made from electrospun nanofibers of CA combined with AuAg nanoclusters. This sensor changes color from red to blue based on the detected levels of Hg^2+^ and Cu^2+^. Furthermore, the sensor leverages RGB analysis via smartphones to accurately quantify the contaminant levels in aquatic products.

### 5.3. Fruit and Vegetable Products

Modern agricultural practices necessitate rigorous monitoring of pesticide residues in horticultural products, with dichlorvos (DZN) representing a high-risk organophosphate insecticide. To address this challenge, Topsoy et al. [66] developed a cost-effective voltammetric sensor through electrospun nanofiber electrodes modified with polycaprolactone and CS. This platform demonstrated reliable DZN detection in tomato matrices at sub-ppm levels, showcasing operational simplicity and field applicability.

The safety testing of processed fruit and vegetable products is equally important. For instance, products with high lipid contents, such as olives and peanuts, can be utilized to produce edible oils, which may also be exposed to chemical contamination during production, storage, and distribution processes. El-Moghazy et al. [67] successfully developed an ultra-sensitive electrochemical biosensor that immobilizes acetylcholinesterase (AChE) within CS/PVA electrospun nanofibers for the rapid detection of methamidophos in olive oil. Due to the spatial structure, high porosity, and large surface area of the electrospun nanofibers, the use of these materials can effectively double the response of the biosensor. The developed biosensor demonstrated excellent performance in detecting methamidophos, with a detection limit of 0.2 nM, significantly lower than the maximum residue limit permitted by international regulations (164 nM).

Mycotoxin contamination poses additional risks in commercial fruits and vegetables. Through the revolutionary combination of nanofibers, electrochemical methods, and aptamer technology, El-Moghazy et al. [55] engineered an innovative electrochemical aptamer-based sensor for detecting ochratoxin A (OTA) in cold brew coffee. The nanofiber-aptamer conjugate exhibited superior analytical performance compared to conventional cast films, demonstrating a dynamic range of 0.002–2 ng mL^−1^, with a detection limit of 0.81 pg mL^−1^. Notably, this system enabled direct OTA in cold brew coffee samples without sample pretreatment, revolutionizing rapid quality control protocols.

### 5.4. Dairy Product

Microbial spoilage constitutes the primary safety concern in dairy systems. Effective and simple testing during the commercial cold chain process is crucial for ensuring the safety of dairy products. Tsai et al. [68] immobilized laccase onto CS/PVA/tetraethylorthosilicate electrospun film (ceCPTL) to develop a time–temperature indicator (TTI) prototype using guaiacol for colorimetric response. In static temperature response tests, the color endpoint of the TTI prototype containing immobilized laccase was capable of responding to a bacterial concentration of 10^6^ colony-forming units (CFU)/mL in milk, with low predictive error. The results of this study indicate that the laccase TTI prototype can serve as a visual monitoring indicator to assist in evaluating the quality of milk within the cold chain.

Currently, research specifically focusing on polysaccharide-based electrospun fibers in the food safety testing of dairy products is limited. However, comparative analyses from other studies can illustrate the effectiveness of food safety testing for dairy products.

Ranjbar et al. [56] have developed a novel electrochemical aptamer sensor for the rapid and sensitive detection of *Staphylococcus aureus* based on a nanocomposite of AuNPs/CNPs/CNFs. This sensor employs electrochemical impedance spectroscopy (EIS), which measures changes in charge transfer resistance across a redox probe on the biosensor, indicating the presence of the target bacteria. This detection method is effective in identifying Staphylococcus aureus in complex solutions and holds significant potential for researching and detecting bacterial contamination in milk. Wang et al. [69] implemented layer-by-layer CA with lysozyme/SA complexes, achieving a significant reduction in Staphylococcus aureus colonies in ultra-high temperature milk at both 4 °C and 25 °C. The nanofiber membranes containing lysozyme ultimately retained Staphylococcus aureus, enabling microbial detection from the membranes and effectively achieving food safety testing outcomes. This method holds significant potential for applications in milk and dairy products.

## 6. Summary and Outlook

Electrospinning is currently one of the techniques for producing sensor materials that is characterized by rapid response times, lightweight construction, and reusability. This review systematically examines electrospinning fundamentals and polysaccharide-based material selection criteria, finding that adjustments to the electrospinning parameters for polysaccharide materials are required to meet the practical application demands. The biocompatibility and biodegradability of polysaccharide materials ensure that these sensors are environmentally friendly and suitable for sustained use in the food industry, meeting the growing demands for sustainable development, particularly for nanofiber applications across meat, aquatic, dairy, and fruit and vegetable sectors. Despite technological advancements, critical challenges require resolution to enable industrial translation:

1. Advanced material characterization: Comprehensive multiscale analysis (e.g., in situ atomic force microscopy [70], X-ray photoelectron spectroscopy [71], and molecular dynamics simulations [72]) is needed to elucidate structure–property relationships governing contaminant–nanofiber interactions. By understanding the principles of these interactions, the development of the detection functions of polysaccharide-based electrospun nanofibers toward similar contaminants can be facilitated. Moreover, this knowledge will optimize their performance in detecting various contaminants.

2. Hybrid sensor architectures: Single-polymer sensors often fail to meet the requirements of practical detection, making the incorporation and cross-linking of multiple materials essential to enhance detection efficacy. For example, the introduction of polyvinyl alcohol and metal ions into electrospun fibers can enhance the mechanical strength of the end products. Combining polysaccharide-based nanofibers with other sensing technologies, such as electrochemical [73] and biosensing, can lead to synergistic effects and improve detection capabilities [74]. This multidisciplinary approach holds the potential to develop more robust and versatile platforms for food safety testing.

3. Smart monitoring systems: Developing systems for real-time monitoring of food safety represents a promising field of exploration. Owing to their high loading capacity and biocompatibility, polysaccharide nanofibers find extensive applications in the food sector. The integration of smart sensing technologies with polysaccharide nanofibers can enhance continuous monitoring of food products throughout the supply chain. Combining sensor technology with a smartphone app to develop a detection system that allows the app to gather and interpret sensor data ensures more accurate safety standards [75]. This approach not only improves real-time responsiveness but also elevates the precision of food safety assessments.

## Data Availability

Data are contained within the article.
